# Electrospun Ni-doped ZnO nanofiber coatings on carbon fabric for enhanced electromagnetic interference shielding

**DOI:** 10.1038/s41598-025-28517-1

**Published:** 2025-12-29

**Authors:** R. Suresha, Jagadeesh R. B. Chandra, Niranjan N. Prabhu, H. K. Sachidananda, Gibin George, Sampath Parasuram, B. Shivamurthy

**Affiliations:** 1 School of Engineering and Information Technology, Department of Electrical & Electronics Engineering, Manipal Academy of Higher Education Dubai International Academic City (DIAC), Dubai Campus. G04, 345050 Dubai, UAE; 2 Department of Electronics & Communication Engineering, Manipal Institute of Technology, Manipal Academy of Higher Education, Manipal, 576104 India; 3 Department of Aeronautical Engineering, Srinivas Institute of Technology, Mangaluru, India; 4 School of Engineering & Information Technology, Department of Mechanical Engineering, Manipal Academy of Higher Education Dubai International Academic City (DIAC), Dubai Campus, G04, 345050 Dubai, UAE; 5 Department of Mechanical Engineering, SCMS School of Engineering and Technology Vidya Nagar, Palissery, Karukutty − 683 576, Ernakulam, Kerala India; 6 Department of Materials Engineering, Indian Institute of Science, Bangalore, India; 7 Department of Mechanical & Industrial Engineering, Manipal Institute of Technology, Manipal Academy of Higher Education, Manipal, 576104 India

**Keywords:** CFRPs, EMI shielding, Electrospinning, Engineering, Materials science, Nanoscience and technology

## Abstract

Electromagnetic interference (EMI) is a critical concern in aerospace and defense applications, where lightweight structural composites must provide effective shielding against high-frequency electromagnetic (EM) radiation. In this work, Ni-doped ZnO (Ni-ZnO) nanofibers were directly deposited onto bidirectional carbon fabric substrates using an electrospinning process, followed by calcination at a high temperature. The nanofiber-coated fabrics were characterized for morphological, structural, and interfacial properties using scanning electron microscopy (SEM), X-ray diffraction (XRD), and Fourier-transform infrared spectroscopy (FTIR). The shielding effectiveness (SE) of the samples was evaluated in the X-band frequency range (8–12 GHz) using a vector network analyzer. The Ni-ZnO nanofiber coating enhanced the absorption-dominated shielding mechanism, resulting in improved attenuation performance compared to unmodified carbon fabric/epoxy laminates. The three-layer Ni-ZnO nanofiber-coated laminate achieved a maximum SE of ~ 85 dB at 10 GHz, corresponding to > 99.99% attenuation of incident EM radiation. This improvement is attributed to synergistic dielectric and magnetic losses, increased interfacial polarization, and enhanced conductive pathways. These findings demonstrate that Ni-ZnO nanofiber-modified carbon fabrics are promising for multifunctional aerospace composite structures requiring both mechanical performance and superior EMI shielding.

## Introduction

Electromagnetic (EM) waves comprise mutually perpendicular electric and magnetic fields that propagate at the speed of light and can originate from both natural and artificial sources. Lightning, thunderstorms, and electrostatic discharges are the natural sources of EM radiation. The artificial sources are communication antennas, control systems, and a wide range of electronic devices that are used in military, industrial, and domestic purposes^1^. Prolonged exposure to EM radiation can cause adverse effects on living organisms. For instance, high-frequency EM radiation can penetrate the skin and damage internal organs^2^. Continuous exposure to non-ionizing radiation in animals can lead to DNA damage^3^, and in plants, it can reduce growth^4^ and cause biochemical alterations, including reduced alanine accumulation^5^. Besides the biological hazards, EM radiation can pose technical risks by interfering with the operation of nearby devices, called electromagnetic interference (EMI). EMI can disrupt the service and affect the accuracy of devices and raise security concerns such as data leakage. To overcome the challenges arising from EM waves, international standards are established, and ANSI set the limit for non-ionizing radiation exposure in 1982, which was revised by IEEE in 1991, followed by U.S. mobile phone radiation regulations in 1997, and ICNIRP guidelines in 1998^6^.

EMI shielding is critical in advanced engineering domains such as aerospace. Metals such as aluminum and ferrous alloys exhibit good EMI shielding properties, but they are rapidly replaced by carbon fiber-reinforced polymer (CFRP) composites due to their superior specific strength, corrosion resistance, fatigue tolerance, and low weight. However, the integration of electronic systems in modern aircraft and spacecraft requires protection from high levels of EM radiation from solar flares, thunderstorms, electrostatic discharges, and radar communication frequencies, especially in the X-band range (8–12 GHz). The same is crucial for radar and satellite-based communication. CFRP composites exhibit good mechanical performance requirements, but their EMI shielding capabilities are inferior to metals. Although CFRPs are conductive and support reflection-dominated shielding, absorption-driven mechanisms are necessary to enhance EMI attenuation and minimize secondary radiation leakage.

Recent advancements in absorption-dominant fabric materials have explored a variety of strategies to enhance electromagnetic wave attenuation through dielectric and magnetic loss mechanisms. For instance, conductive polymer composites, metal oxide-infused textiles, and hybrid nanostructured fabrics have shown promising results in achieving high shielding effectiveness with minimal reflection. Baheti et al^7^. developed a layered textile combining activated carbon and copper-plated cotton to enhance EMI shielding and electrothermal performance. Their design achieved ~ 32 dB shielding and rapid heating, offering scalable solutions for smart fabrics and wearable electronics. Similarly, Singh et al^8^. investigated the EMI shielding and joule heating performance of woven and nonwoven activated carbon fabrics derived from Kevlar fibers. Their study found that nonwoven structures carbonized at 1000 °C achieved superior conductivity, shielding effectiveness (~ 26.9 dB), and surface temperature (~ 180 °C at 5 V) compared to woven fabrics. In another study, Sun et al^9^. designed a flexible textile integrating Ag nanowires and hollow Fe₃O₄@NC nanospheres, achieving high EMI shielding (~ 50.1 dB) with low reflectance (~ 2.6 dB). Their absorption-dominant structure enables “absorption, reflection, and reabsorption” of electromagnetic waves, ideal for wearable shielding applications. These approaches collectively emphasize the significance of material selection, nano structuring, and hybridization in developing fabrics with absorption-dominant EMI shielding capabilities.

Many reports in the literature attempted to tailor CFRP composites to enhance absorption-dominated shielding by introducing dielectric and magnetic loss mechanisms through structural modification and nanoscale functionalization. The EMI shielding performance is mainly dependent on laminate architecture and fiber orientation. Gupta et al^10^. reported that unidirectional carbon fiber laminates fabricated by hot compression molding exhibited significant absorption in the X-band, although reflection still dominated. Incorporating electrospun nanofiber membranes, nanoparticle coatings, and hybrid nanostructures onto carbon fabrics improved the performance of CFRPs in EMI shielding. For instance, Anwar et al^11^. demonstrated that Fe_3_O_4_, graphene-, carbon-, and polypyrrole-based electrospun nanofiber membranes achieved a shielding effectiveness (SE) of 28.4 dB at 3 mm thickness. This composite formulation absorbs 99.7% of the radiation through magnetic loss, interfacial polarization, and multiple scattering. Similarly, Bayat et al^12^. embedded Fe_3_O_4_ nanoparticles in carbon nanofiber mats, achieving up to 67 dB SE in the X-band at ~ 0.7 mm thickness, driven by dielectric and magnetic loss in the presence of Fe_3_O_4_. Other approaches, such as Fe_3_O_4_ hollow spheres^13^ and nanoparticle synthesis methods^14^, have highlighted the role of morphology, crystallinity, and purity in tailoring magnetic and dielectric responses of the CFRPs for effective EMI attenuation.

Nickel (Ni), zinc oxide (ZnO), and carbon fibers were selected based on their complementary electromagnetic characteristics and structural compatibility. Ni exhibits high magnetic permeability and natural resonance behavior, which introduces significant magnetic loss and facilitates the attenuation of incident electromagnetic waves through magnetic dipole relaxation^15^. ZnO, a wide bandgap semiconductor with high dielectric constant, enhances dielectric polarization and interfacial charge storage, promoting absorption rather than reflection^16^. Meanwhile, carbon fiber offers high electrical conductivity and structural integrity, serving as a lightweight conductive backbone for reflection-based shielding and mechanical reinforcement. The synergistic combination of these three components enables a hybrid dielectric-magnetic system with improved impedance matching and absorption-driven EMI shielding efficiency.

In the present study, novel Ni-ZnO nanofiber-coated carbon fabrics are prepared via electrospinning and subsequently integrated into CFRP composites. While previous studies have investigated Fe₃O₄, graphene, and conductive polymer nanofibers for EMI shielding, no prior work has reported the use of Ni-ZnO hybrid nanofibers integrated with CFRPs for absorption-dominated EMI attenuation in the X-band region. Unlike Fe_3_O_4_/carbon systems, where shielding is primarily governed by magnetic loss and interfacial reflection, the proposed Ni-ZnO/carbon system exhibits a synergistic dielectric-magnetic coupling mechanism. Here, ZnO contributes to dielectric loss through dipolar and interfacial polarization, whereas Ni enhances magnetic and conduction losses, resulting in a balanced and absorption-driven EMI shielding mechanism. Furthermore, the nanofiber architecture enables uniform nanoscale coating, enhanced interfacial bonding, and improved charge transport, leading to superior absorption efficiency and mechanical integrity compared to Fe_3_O_4_/carbon counterparts. The objective of this study is to investigate the EMI shielding performance of Ni-ZnO nanofiber-CFRP laminates in the X-band frequency range, with particular emphasis on their mechanistic distinction and absorption-dominated shielding behavior suitable for aerospace applications.

Compared to previous studies involving Fe₃O₄, graphene, and polypyrrole-based nanofiber systems, the present work introduces a unique combination of Ni and ZnO nanofibers integrated into CFRP laminates. Liu et al. developed Fe₃O₄/PPy textiles with up to 47 dB shielding and high durability^17^. Xing et al. demonstrated PPy/Fe₃O₄/graphene composites with ~ 66 dB reflection loss at 14.88 GHz^18^. In contrast, the Ni-ZnO system leverages both dielectric and magnetic losses, enabling absorption-dominant shielding with improved impedance matching. Merizgui et al. explored Ni-ZnO-based chopped carbon fiber composites, but their approach lacked the nanofiber architecture and integration into aerospace-grade CFRPs^19^. To our best knowledge, this is the first report demonstrating the synergistic EMI attenuation behavior of Ni-ZnO nanofiber-coated carbon fabrics within CFRP laminates, specifically tailored for X-band aerospace applications.

## Materials and methods

### Materials

A bi-directional, plain-weave carbon fabric (supplied by M/s. Bhor Chemical & Plastics Pvt. Ltd., Nasik, India; Batch No. W21A31/3298) was used as the reinforcement. The fabric possessed a 160 g per square meter (GSM) and consisted of 3 K carbon fibres. Each filament had an approximate diameter of ~ 7 μm, and the bundle contained ~ 3000 filaments per tow. The nominal density of the carbon fibers was ~ 1.8 g/cm³, and the fabric exhibited low porosity suitable for resin impregnation. These characteristics ensure high tensile strength and dimensional stability, making it ideal for aerospace-grade CFRP laminates. Poly (vinyl alcohol) (PVA) (Synthesis grade; molecular weight, $\:\stackrel{-}{{M}_{w}}$=115,000 g·mol⁻¹) was procured from M/s. Loba Chemicals Pvt. Ltd., Mumbai, India. Zinc acetate dihydrate (ZnAc₂) and Nickel (II) acetate tetrahydrate (NiAc_2_) were obtained from M/s. Sigma Aldrich. Bisphenol A epoxy resin with suitable hardener was used as matrix material. Laboratory-grade acetone and deionized water were employed for washing and solution preparation. All chemicals and materials were used as received without any purification.

### Synthesis of Ni-ZnO nanofiber-coated carbon fabric

**Fig. 1:**
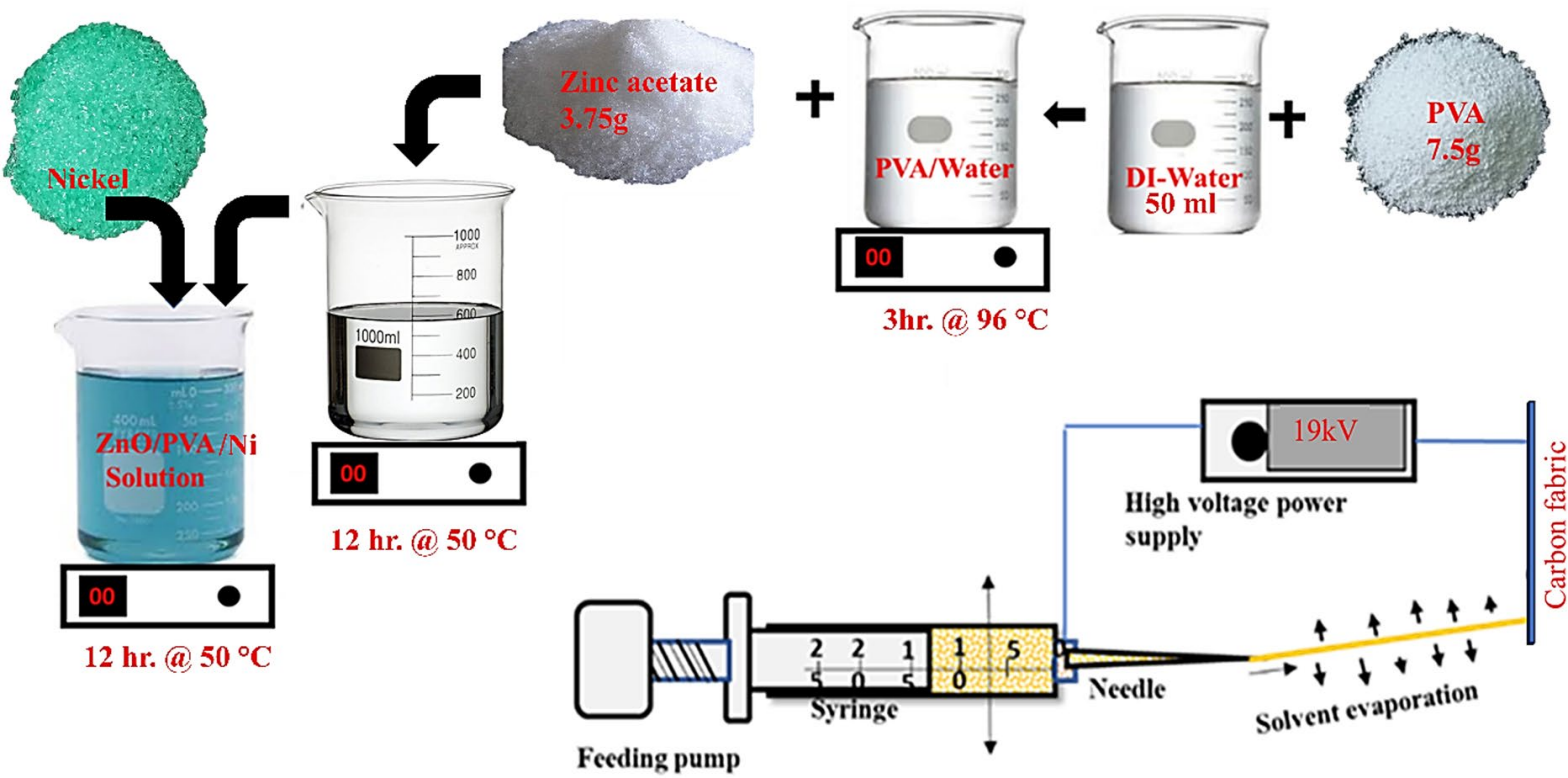
Precursor solution preparation and synthesis of nanofiber on carbon fabric substrate.

Figure 1 illustrates the sequential steps involved in preparing the Ni-ZnAc_2_/PVA precursor solution for nanofiber synthesis on carbon fabric. Initially, 7.5 g of polyvinyl alcohol (PVA) was dissolved in 50 mL of deionized water and stirred for 3 h at 96 °C. Subsequently, 7.5 wt% zinc acetate (ZnAc₂) was added to the PVA solution, followed by continuous stirring for 12 h at 60 °C. This composition was chosen based on optimized parameters reported in the literature^20^. Finally, 2 wt% NiAc_2_ was introduced into the prepared PVA/ZnAc₂ solution and stirred for an additional 12 h at 60 °C to obtain the final electrospinnable solution. The Ni doping concentration was fixed at 2 wt% based on both preliminary optimization trials and literature reports, where similar Ni contents (1–3 wt%) have been found to yield enhanced structural and functional properties of ZnO nanofibers without secondary phase formation^21,22^. In our synthesis, higher Ni precursor concentrations resulted in unstable electrospinning and irregular fiber morphology, whereas 2 wt% NiAc₂ provided uniform, continuous nanofibers with good reproducibility.

**Fig. 2:**
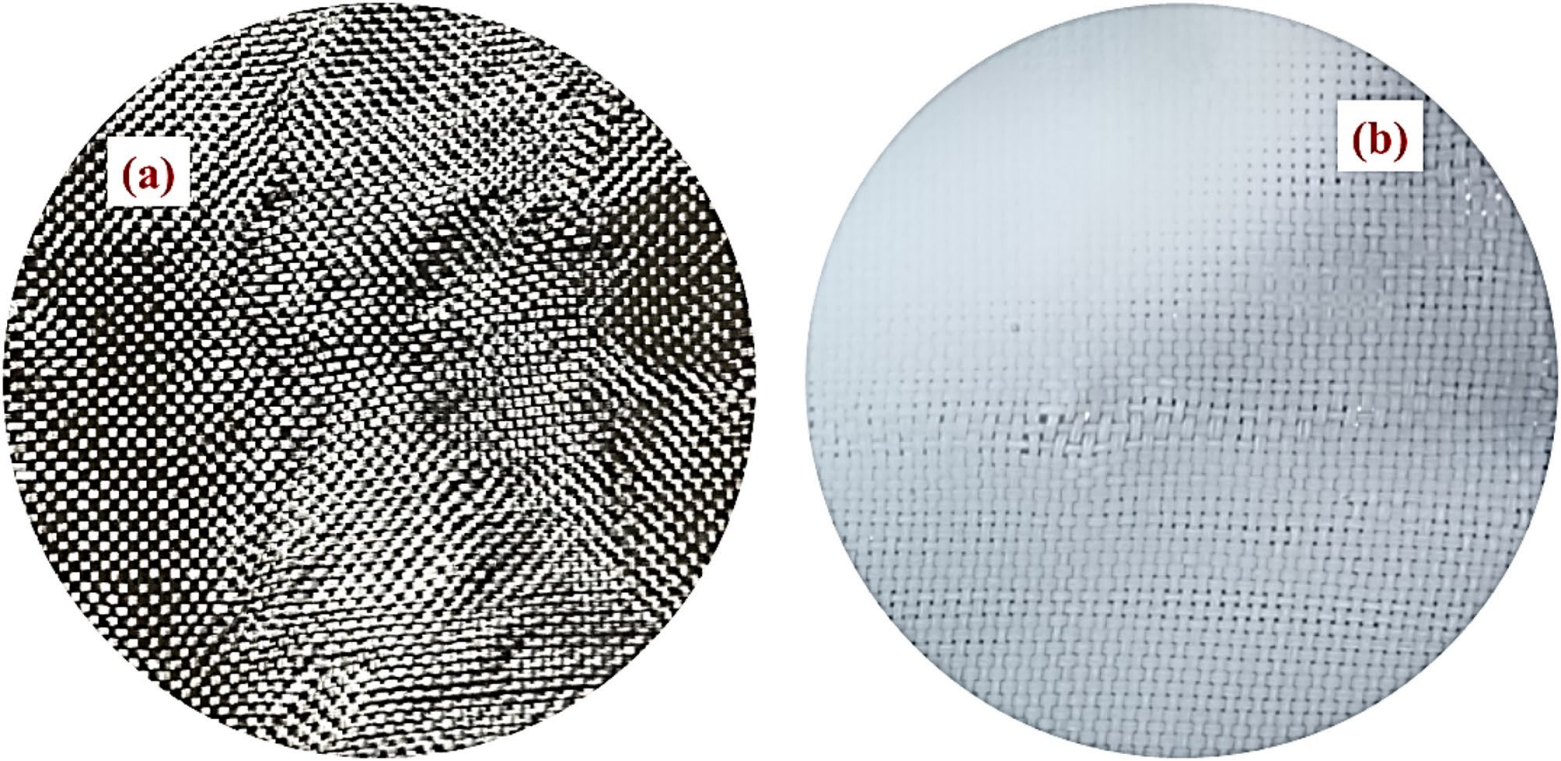
Digital photograph of (**a**) Neat carbon fabric and (**b**) Nanofiber deposited on carbon fabric.

The Ni-ZnAc_2_/PVA precursor nanofiber on carbon fabric substrate was synthesised by the electrospinning process. The schematic diagram of the process is mentioned in Fig. 1. To deposit the fibers on the carbon fabric, a suitable size of carbon fabric was cut and cleaned with acetone, further cleaned by deionised water, and dried in a hot air oven at 60 °C for about 1 h and mounted on the collector of the electrospinning machine. The prepared Ni-ZnAc_2_/PVA precursor solution was loaded in a 2.5 ml syringe with a needle. The inner diameter of the needle is 0.5 mm, and the length is 20 mm. Further, the syringe needle was connected to the positive terminal of a DC voltage of 19 kV and maintained a 20 cm distance from the collector. The high voltage connected to the needle overcomes the surface tension of the solution in the syringe needle, and a charged precursor jet solution gets ejected from the needle and deposited in the form of precursor composite nanofiber on a carbon fabric substrate. The electrospinning process employed an applied voltage of 19 kV and a needle-to‐collector distance of 20 cm, which were selected based on our prior optimization study of the PVA/ZnAc₂ precursor solution^23^. In that work we systematically varied applied voltage (16–19 kV) and spinneret to needle distance (12–20 cm) and observed that 19 kV/20 cm produced continuous, bead‐free nanofibers with uniform deposition. The digital photograph of neat carbon fabric, and Ni-ZnAc_2_/PVA nanofibers deposited on carbon fabric is shown in Fig. 2 (a & b), respectively. The Ni-ZnAc_2_/PVA nanofibers on the carbon fabric were subjected to a calcination process at 480 °C and completely removing the PVA from the nanofibers. The present work optimized the Ni-ZnAc₂/PVA precursor composition; therefore, a control sample of pure ZnO nanofibers was not carried out. However, the observed increases can be attributed to Ni incorporation. Similar effects of Ni doping on ZnO have been widely reported in the literature^21,24^, supporting the inference that Ni acts as an active dopant modifying the structural characteristics of ZnO nanofibers.

### Fabrication of composite laminate

**Fig. 3:**
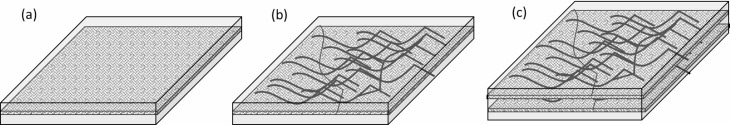
The architecture of the laminates (**a**) CF/E, (**b**) CF/E + NF, and (**c**) CF/E + NF + NF + CF/E.

The carbon fabric with Ni-ZnO nanofibers on its surface was used to fabricate CFRP composites by the layup method. The architecture of layers and laminates prepared by this process was designated and listed as shown in Fig. 3; Table 1.

**Table 1:** The architecture of layers and laminates prepared.

Sl. No.	Architecture of laminate	Sample designation
1	Neat carbon fabric/epoxy	CF/E
2	1-layer carbon fabric/epoxy with 1-layer Ni-ZnO nanofibers	CF/E + NF
3	Two-layers carbon fabric/epoxy with 2-layers Ni-ZnO nanofibers	CF/E + NF + NF + CF/E

### Characterization

ZnAc_2_-PVA-Ni nanofibers were deposited on carbon fabric using the electrospinning technique. The morphology of the deposited nanofibers was examined using a scanning electron microscope (Carl Zeiss, Germany). SEM images were analyzed with ImageJ software to determine the average fiber diameter, size distribution, and structural characteristics. The structural analysis of the sample was investigated by X-ray powder diffraction (XRD, Rigaku Mini Flex 600, Japan) with angles from 10° to 70° at a scanning speed of 0.5° m^− 1^. The interfacial bonding between the nanofibers and carbon fabric was analyzed through FTIR spectroscopy. The electromagnetic shielding effectiveness of neat carbon fabric and nanofiber-deposited carbon fabric laminate samples was measured using a Vector Network Analyzer (VNA), following ASTM D4935 standards. The Coaxial Transmission Line Method (CTLM) was employed to determine reflected, absorbed, and transmitted components. The scattering parameters S11 (reflection coefficient) and S21 (transmission coefficient) were used to calculate SE_R_ ​ and SE_A_ using equations.

The total shielding effectiveness is given by the equation.

1 $$SE_{T} = {\text{ }}SE_{R} + SE_{A}$$

Where, SE_T_ is total shielding effectiveness (dB), SE_R_ is shielding effectiveness due to reflection (dB) and SE_A_ is shielding effectiveness due to absorption (dB).

Since the multiple reflection term (SE_M_) is negligible in the high-frequency range, it was excluded from the final SE calculation.

## Results and discussion

### Morphology of nanofibers

**Fig. 4:**
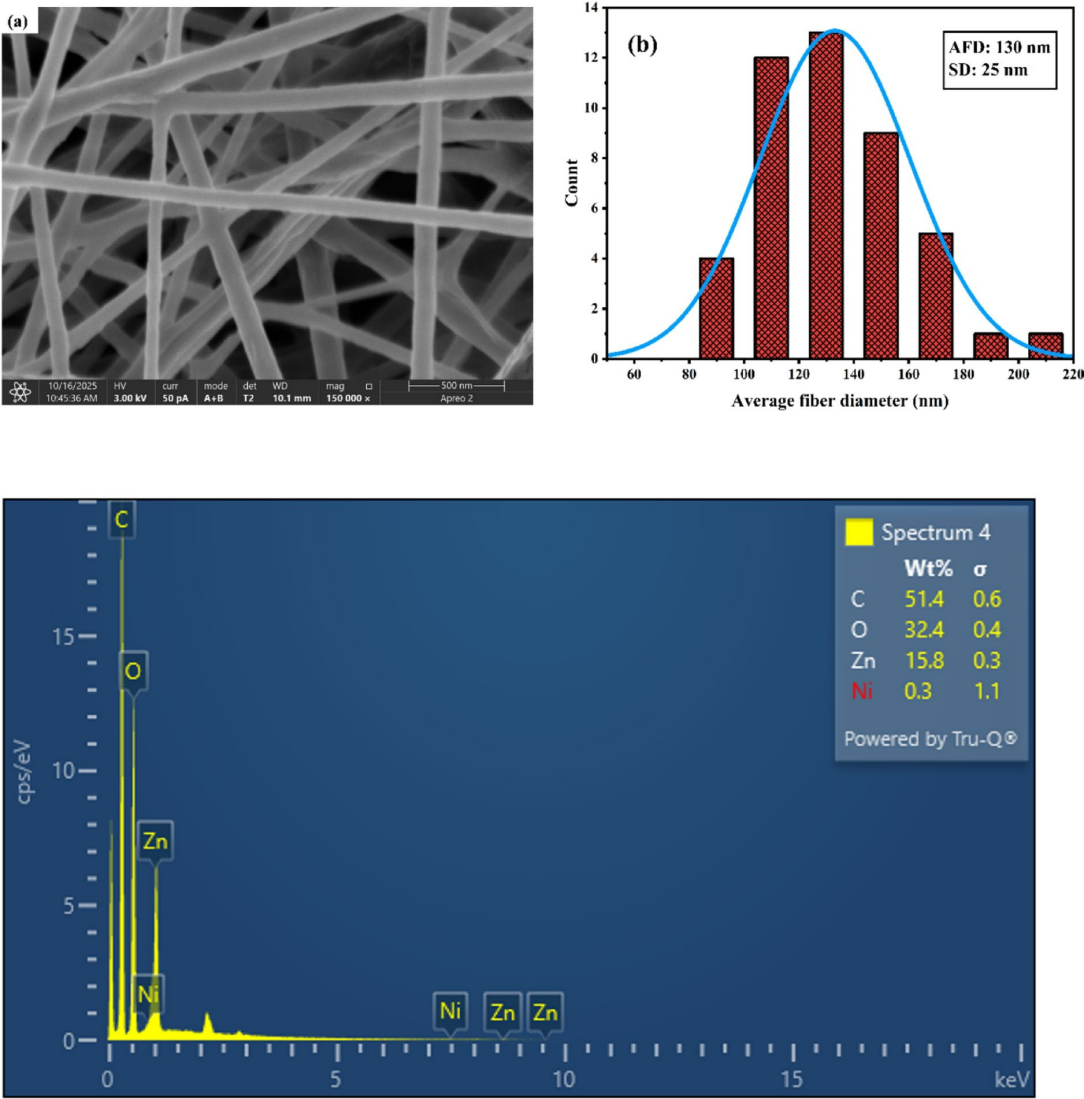

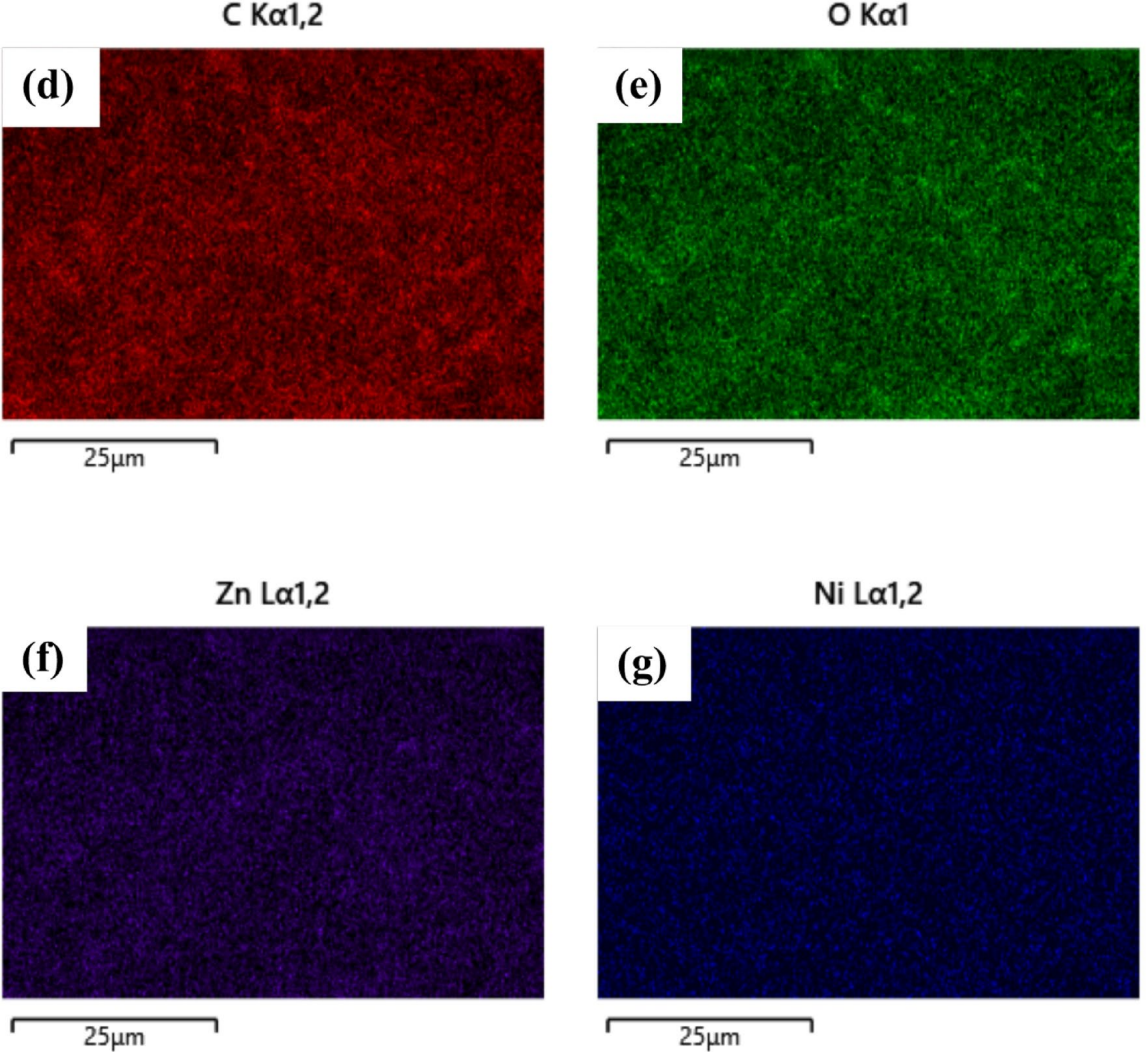
(**a**) FESEM image of nanofibers and (**b**) average fibre size and size distribution (**c**) EDS spectrum of Ni–ZnO nanofiber-coated carbon fabric confirming the presence of Zn, Ni, O, and C elements (**d**-**g**) Elemental mapping of (**d**) C, (**e**) O, (**f**) Zn, and (**g**) Ni on Ni–ZnO nanofiber-coated carbon fabric, showing uniform distribution of ZnO and dispersed Ni doping.

The ZnAc₂-PVA-Ni nanofibers deposited on carbon fabric were dried at 55 °C under vacuum for 2.5 h, followed by pyrolysis at 480 °C. The surface morphology was examined using a scanning electron microscope (Carl Zeiss, Germany). Figure 4(a) shows the FESEM image of the nanofiber-coated carbon fabric, revealing uniform nanofiber distribution with diameters ranging from 80 to 220 nm. Minor droplet formations were observed, attributed to variations in precursor viscosity. The average fiber diameter, determined through ImageJ analysis, was approximately 130 nm, and the corresponding diameter distribution is presented in Fig. 4(b).

To confirm the elemental composition, EDS analysis was performed, which verified the presence of Ni and Zn, along with oxygen, indicating the successful formation of Ni-doped ZnO nanofibers. Figure 4 (c) shows the EDS spectrum of the Ni–ZnO nanofiber-coated carbon fabric. The analysis confirms the presence of Zn (15.8 wt%), Ni (0.3 wt%), oxygen (32.4 wt%), and carbon (51.4 wt%). The relatively low Ni content aligns with the optimized precursor composition (2 wt% NiAc₂), ensuring uniform doping without the formation of secondary phases. These results validate the successful synthesis of Ni-doped ZnO nanofibers on the carbon fabric substrate. Figures 4 (d–g) present the elemental mapping of C, O, Zn, and Ni on the nanofiber-coated carbon fabric. Carbon is uniformly distributed, corresponding to the fabric substrate. Oxygen and zinc show homogeneous dispersion, confirming the formation of ZnO nanofibers. Nickel appears as fine, evenly distributed spots, consistent with the optimized doping level (0.3 wt% as determined by EDS analysis). This uniform elemental distribution supports the structural integrity and functional design of the coated fabrics for absorption-dominated EMI shielding.

### X-ray diffraction study

**Fig. 5:**
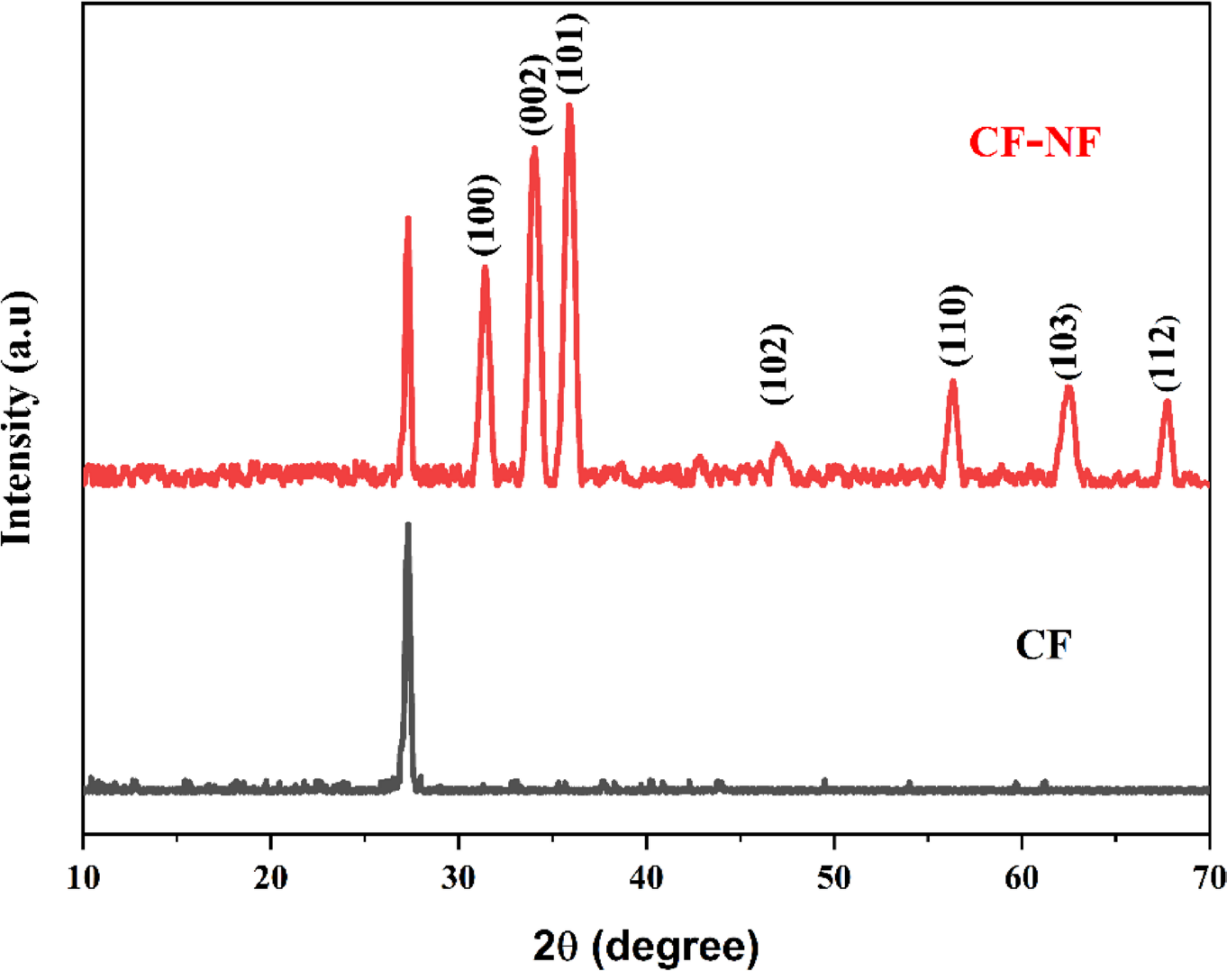
XRD pattern of carbon fabric and Ni-ZnO nanofiber-coated carbon fabric.

The crystalline structure of Ni-ZnO nanofibers on carbon fiber was calculated using the XRD pattern shown in Fig. 5. The diffraction planes of Zinc oxide nanoparticles can be assigned as (100), (002), (101), (102), and (110) of pure hexagonal wurtzite phase. The diffraction peaks of Ni-ZnO are close to the ICDD file No: 89–1397. No secondary phase was observed in the pattern, confirming the successful doping of Nickel into ZnO. The crystalline size of synthesized compositions was computed from the full width at half maximum (FWHM) of all peaks (100), (002), (101), (102), (110), (103), (200), (112) and (201) by using the Debye Scherrer’s equation.

2 $$D\: = \frac{{n\lambda \:}}{{\beta \:\cos \theta \:}}$$

Where ‘λ’ is the X-ray wavelength (λ = 1.5418 × 10^− 10^ Å), ‘n’ is the Scherrer constant value taken as 0.9, ‘β’ is the FWHM in radians, and θ is the Bragg’s angle. The crystallite size of the Ni- ZnO nanofibers is found to be 23 nm. The lattice parameters a and c are found to be 3.2 Å and 5.21 Å, matching with the standard ZnO values.

### FTIR analysis

**Fig. 6:**
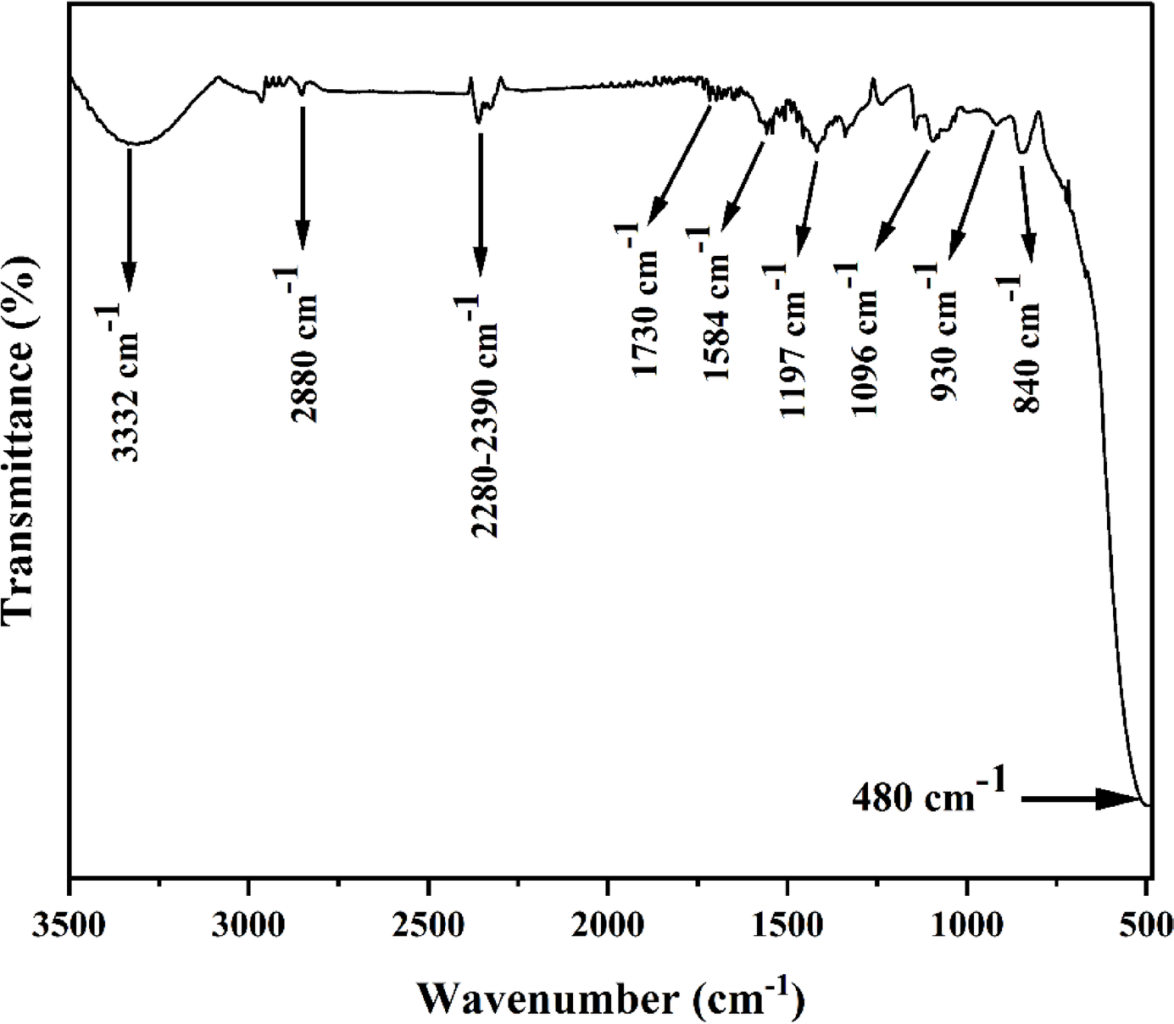
FTIR spectra of Ni-CF.

To identify the functional groups present in the CF–NF (nanofiber synthesized on carbon fabric), FTIR spectroscopy was performed, and the results are shown in Fig. 6. The broad absorption band around 3330 cm⁻¹ corresponds to O–H or N–H stretching vibrations, indicating the presence of oxygen- or nitrogen-containing functionalities on the carbon surface^25^. The peak at approximately 2880 cm⁻¹ is attributed to C–H stretching vibrations from aliphatic groups^26^. The absorption peaks observed at 1730 cm⁻¹ and 1584 cm⁻¹ correspond to C═O and aromatic C═C stretching vibrations, respectively^27^. The band at 1197 cm⁻¹ is assigned to phenolic C–OH bending, while the peaks at 1096 cm⁻¹ and 930 cm⁻¹ are associated with C–O–C stretching modes^28^. The peak at 840 cm⁻¹ is ascribed to aromatic C–H out-of-plane bending vibrations, and the strong band centered near 480 cm⁻¹ corresponds to Zn-O and Ni–O bond vibrations, indicating the integration of Ni-ZnO nanofibers in the carbon fiber^29^.

### Electromagnetic shielding effectiveness

Thickness and weight of all the laminates were measured accurately, and density of the laminates were estimated. It was found that the density of the laminates was in the range of 1.45–1.55 g/cm³. The nanofiber wt% loading on each laminate was approximated based on the volume of precursor solution used for nanofiber deposition, weight of fabric and matrix used for the laminates. These physical parameters were used to calculate the specific shielding effectiveness (SSE) to enable a normalized comparison of intrinsic electromagnetic wave attenuation. The measured and estimated physical properties of laminates are summarized in Table 2.

**Table 2:** Thickness, density and nanofiber concentration of the CFRP laminates.

Sample	Thickness (mm)	Density (g/cm³)	Estimated NF (wt%)
CF/E	0.40	1.50	0
CF/E+NF	0.48	1.51	0.5-1.0
CF/E+NF+NF+CF/E	0.75	1.52	1.0-2.0

**Fig. 7:**
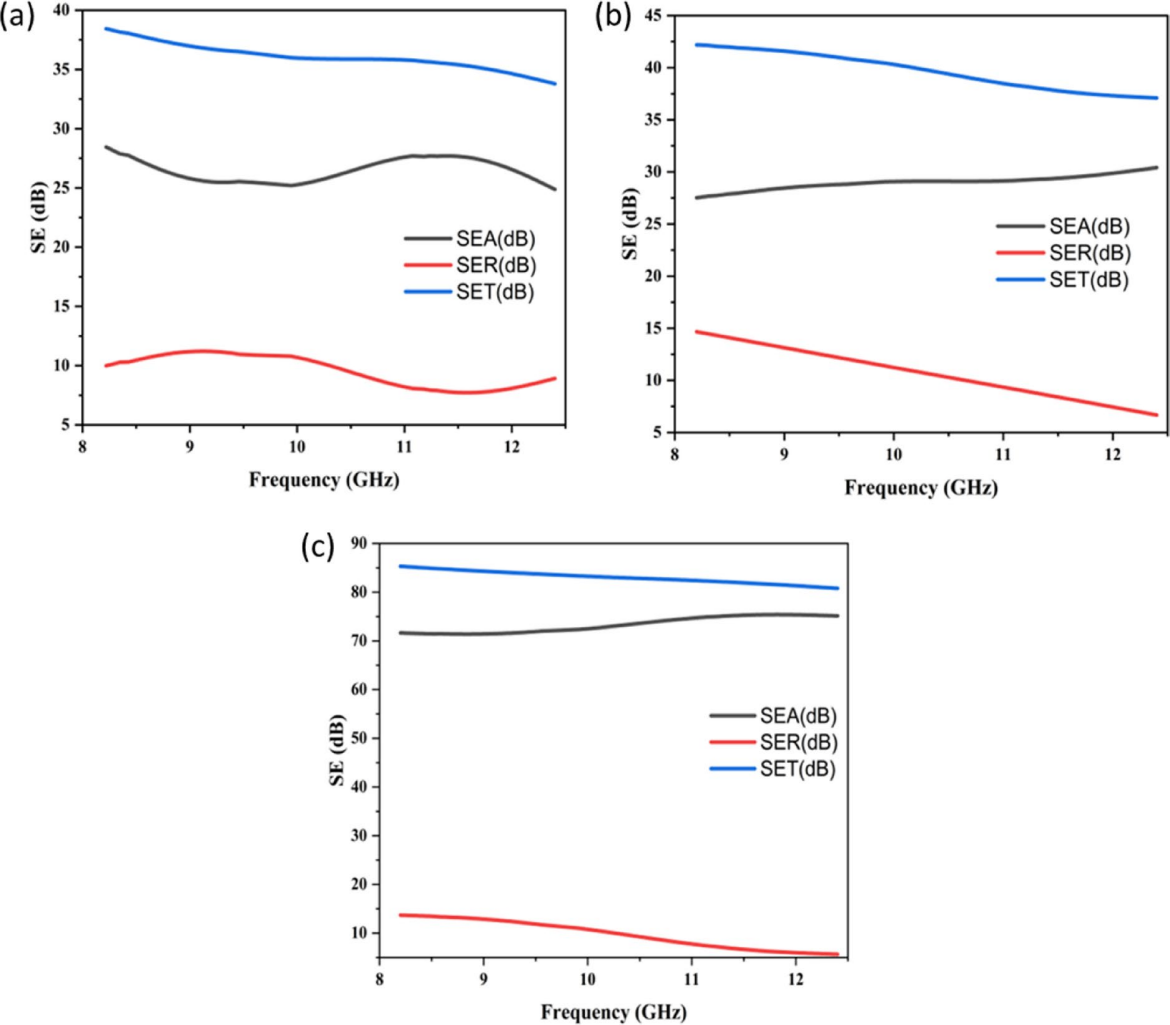
Electromagnetic shielding effectiveness of the prepared Ni-ZnO/CFRP composite laminates (**a**) CF/E, (**b**) CF/E + NF, and (**c**) CF/E + NF + NF + CF/E.

Figure 7 compares the EMSE of the nanofiber-incorporated composites to evaluate their ability to protect against EMI. Measurements across the X-band frequency range indicated that CF/E exhibited the lowest shielding effectiveness (SE_T_) of ~ 35–38 dB, mainly contributed by absorption (SE_A_ ≈ 23–27 dB) and a smaller portion from reflection (SE_R_ ≈ 8–12 dB). The CF/E + NF composites (Fig. 7b) demonstrated enhanced performance compared to CF/E, maintaining consistent shielding across the tested range and offering a balanced level of conductivity and absorption, reaching SE_T_ values of ~ 38–42 dB across the band. From the results, it can be observed that SE_A_ increased to ~ 27–30 dB, while SE_R_ decreased from ~ 15 dB at 8 GHz to ~ 5 dB at 12 GHz, indicating a stronger dominance of absorption over reflection. The multilayer configuration (CF/E + NF + NF + CF/E, Fig. 7c) achieved the highest performance, with SE_T_ values of ~ 82–85 dB, dominated by absorption (SE_A_ ≈ 70–75 dB) and only minor reflection (SE_R_ ≈ 12 dB at 8 GHz, dropping to ~ 5 dB at 12 GHz). The CF/E + NF + NF + CF/E outperformed both CF/E and CF/E + NF composites, achieving the highest shielding effectiveness throughout the frequency spectrum.

The electromagnetic shielding performance results clearly indicate a progressive enhancement when Ni-ZnO nanofibers are incorporated into carbon fabric/epoxy composites, and this improvement is further enhanced through multilayer stacking. The neat carbon fabric exhibits moderate shielding effectiveness (SE), primarily due to its intrinsic electrical conductivity, with minimal absorption contribution. Introducing a single layer of Ni-ZnO nanofibers significantly boosts SE through the synergistic effects of dielectric loss from ZnO nanoparticles, magnetic loss from Ni atoms, and enhanced multiple internal reflections resulting from the nanofiber surface morphology. In the two-layer configuration, SE improves further because stacked interfaces increase the interaction path length for electromagnetic waves, thereby amplifying attenuation via multiple scattering and combined dielectric–magnetic absorption mechanisms.

To further support the absorption-dominant shielding behavior, a quantitative analysis of the electromagnetic loss mechanism was carried out using the measured S-parameters. The total shielding effectiveness (SE_T_), reflection (SE_R_), and absorption (SE_A_) components were determined using:

3 $$\:S{E}_{T}=-10{\mathrm{l}\mathrm{o}\mathrm{g}}_{10}(\mid\:{S}_{21}{\mid\:}^{2}),S{E}_{R}=-10{\mathrm{l}\mathrm{o}\mathrm{g}}_{10}(1-\mid\:{S}_{11}{\mid\:}^{2}),S{E}_{A}=S{E}_{T}-S{E}_{R}$$

The results reveal that more than 80–90% of the total shielding in Ni–ZnO/CFRP laminates arises from absorption, confirming the absorption-driven nature of the EMI shielding.

In addition, the attenuation constant (α), which quantifies the material’s ability to dissipate incident electromagnetic energy, was estimated using:

4 $$\:\alpha\:=\frac{1}{d}\mathrm{l}\mathrm{n}(\frac{(1-R{)}^{2}}{2T}+\sqrt{(\frac{(1-R{)}^{4}}{4{T}^{2}}+{R}^{2})})$$

where *R = |S₁₁|²*, *T = |S₂₁|²*, and *d* is the sample thickness. The Ni-ZnO nanofiber-reinforced composites exhibit markedly higher α compared to unmodified CFRP, due to the synergistic dielectric-magnetic loss contributions of the Ni-ZnO heterostructure and improved impedance matching^30^.

Such improvements are consistent with prior studies where hybrid dielectric-magnetic fillers demonstrated superior EMI shielding through synergistic polarization and magnetic resonance effects^12,31^.

To provide a normalized evaluation of shielding performance, the specific shielding effectiveness (SSE) was calculated using the relation:

5 $$\:SSE=\frac{S{E}_{T}}{\rho\:\cdot\:t}$$

where $\:S{E}_{T}$is the total shielding effectiveness (dB), $\:\rho\:$ is the laminate density (g/cm³), and $\:t$ is the laminate thickness (cm). The calculated specific shielding effectiveness (SSE) are presented in Table 3.

**Table 3:** Specific EMI shielding effectiveness (SSE) of CFRP laminates.

Sample	SET (dB)	ρ (g/cm³)	t (cm)	SSE (dB·cm²/g)
CF/E	36	1.5	0.040	600
CF/E + NF	40	1.51	0.048	551
CF/E + NF + NF + CF/E	84	1.52	0.075	737

The SSE results demonstrate that the multilayer Ni-ZnO nanofiber-modified laminate exhibits the highest intrinsic shielding efficiency, confirming that its enhanced performance originates from synergistic dielectric–magnetic loss mechanisms rather than thickness alone.

Similar trends have been reported for Ni-ZnO-carbon systems, in which the magnetic component enhances absorption while the dielectric component increases interfacial polarization^32^. Pande et al^33^. fabricated layered composites of multi-walled carbon nanotubes dispersed in a polymethyl methacrylate (MWCNT-PMMA) matrix and investigated their EMI shielding performance in the X-band. They showed that stacking multiple thin films enhanced overall shielding effectiveness to ~ 40 dB, mainly due to absorption from multiple internal reflections, while reflection contributions remained nearly constant. Similarly, Singh et al^34^. reported that increasing rGO content enhanced shielding effectiveness due to improved electrical conductivity and interconnected networks, with absorption as the dominant attenuation mechanism. This collective evidence underscores that Ni-ZnO nanofiber-modified carbon fabrics achieve superior shielding by leveraging multi-mechanism loss processes and optimized layer structuring. The improved absorption-dominant EMI shielding performance of the Ni-ZnO nanofiber-coated CFRP composite arises from the synergistic interaction between magnetic Ni nanoparticles and dielectric ZnO within the conductive carbon fabric matrix. The Ni-ZnO heterointerface promotes interfacial polarization and multiple loss mechanisms (magnetic and dielectric), while the carbon fabric facilitates electron transport and multiple internal reflections. This hierarchical structure enables efficient attenuation of incident microwaves through absorption rather than reflection, which is advantageous for aerospace and stealth applications.

Similar heterostructure-based absorption enhancement has been recently reported by Jia et al^30^., where hybrid magnetic-dielectric interfaces significantly improved impedance matching and absorption. However, the present study uniquely integrates Ni-ZnO hybrid nanofibers directly onto carbon fabrics, establishing a flexible and lightweight EMI shielding composite with superior absorption-to-reflection ratio, distinguishing it from previously reported Fe₃O₄/carbon systems.

The EMI shielding performance of the composites is strongly influenced by both electrical conductivity and laminate thickness. Neat CF/E laminates exhibit moderate conductivity, resulting in SE_T_ values of ~ 35–38 dB dominated by reflection. Incorporation of Ni-ZnO nanofibers enhances surface conductivity and introduces dielectric–magnetic loss mechanisms, improving impedance matching and promoting absorption. The single-layer CF/E + NF laminate, with an estimated thickness of ~ 480 μm, achieves SE_T_ values of ~ 38–42 dB, while the multilayer CF/E + NF + NF + CF/E laminate, with a thickness of ~ 746 μm, reaches ~ 82–85 dB. This progressive improvement is attributed to increased conductive pathways and longer propagation paths for EM waves, enabling multiple scattering and interfacial polarization. Similar trends have been reported in literature^35–37^, where higher conductivity and thickness synergistically enhance EMI attenuation. These results confirm that optimizing both conductivity and structural thickness is critical for achieving superior absorption-dominated shielding in hybrid CFRP composites.

The absorption-dominated shielding behavior observed in Ni-ZnO nanofiber-coated CFRP laminates can be directly correlated with their physical and morphological features. FESEM analysis revealed a uniform nanofiber network, which promotes multiple scattering and interfacial polarization. Elemental mapping confirmed homogeneous dispersion of Ni and ZnO, enabling synergistic dielectric-magnetic coupling and improved impedance matching. These structural attributes minimize reflection and maximize absorption, as evidenced by SEA values exceeding 70 dB in multilayer configurations. Furthermore, the coated carbon fabrics remain lightweight and flexible, adding negligible composite exhibit weight to CFRP laminates while preserving handling characteristics. This combination of high absorption efficiency, low weight, and flexibility makes the proposed composites highly suitable for aerospace, stealth, and wearable EMI shielding applications.

### Influence of electrical and magnetic properties on EMI shielding mechanism

The electromagnetic shielding behavior of the nanofiber-reinforced CFRP laminates can be comprehensively interpreted using established electromagnetic principles and representative literature trends for Ni-ZnO-carbon systems^38,39^. The synergetic influences of electrical conductivity, dielectric loss, magnetic loss, impedance matching, and multilayer-induced multiple scattering govern the observed SE_T_, SE_A_, and SE_R_ trends.

The neat CF/E laminate exhibits moderate shielding effectiveness (~ 35–38 dB), primarily governed by reflection. This behavior arises from intrinsic electrical conductivity of carbon fabric. The **c**arbon fabrics has moderate conductivity, leading to a significant impedance mismatch with free space. This mismatch reflects a large fraction of the incident electromagnetic wave, contributing to the measured SE_R_ (~ 8–12 dB). In the absence of high-loss phases, the material dissipates limited electromagnetic energy internally, resulting comparatively lower SE_A_ values. Although the woven geometry in the fabric provides some internal scattering, attenuation remains modest. Consequently, the neat laminate demonstrates reflection-dominated EMI shielding.

The introduction of Ni–ZnO nanofibers on carbon fabric in case of Ni–ZnO nanofiber-coated laminates (CF/E + NF), it was significantly enhanced absorption and overall shielding effectiveness due to the; (i) dielectric loss from ZnO, (ii) magnetic loss from Ni and (iii)improved impedance matching. ZnO contributes dipolar and interfacial polarization as well as defect-related relaxation phenomena, increasing the dielectric loss factor (ε″) and promoting energy dissipation. Nickel introduces eddy-current loss, natural magnetic resonance, and domain-wall motion, elevating magnetic loss. These mechanisms correlate with the increased SE_A_ (~ 27–30 dB) observed for CF/E + NF. Furthermore, the hybrid dielectric–magnetic coating reduces surface impedance mismatch, allowing more electromagnetic energy to enter and be absorbed within the laminate. This is consistent with the reduction in SE_R_ from ~ 15 dB at 8 GHz to ~ 5 dB at 12 GHz. Also, the nanofiber network introduces numerous interfaces, extending the propagation path and enhancing absorption. As a result, CF/E + NF exhibits absorption-dominated shielding with balanced dielectric and magnetic loss contributions.

In case of CF/E + NF + NF + CF/E laminates, the multilayer configuration achieves the highest shielding performance (~ 82–85 dB), with more than 80–90% of total SE originating from absorption (SE_A_ ≈ 70–75 dB). This superior efficiency arises from (i) synergistic dielectric–magnetic loss (ii) enhanced multiple reflections and (iii) reduced skin depth.

The double-stacked Ni-ZnO nanofiber layers enhance both ε″ and µ″, intensifying microwave attenuation. Each layer interface acts as a scattering dominating, prolonging the electromagnetic propagation path and strengthening attenuation. Also, higher effective µ and σ values reduce δ in Ni-ZnO coated laminates, enabling rapid dissipation of electromagnetic energy within a shallow region of the composite. Furthermore, the multilayer stacking increases the interaction volume for EM waves, amplifying absorption. These combined effects result in highly efficient absorption-based EMI shielding, making the multilayer Ni-ZnO nanofiber-coated laminate the best performing configuration.

## Conclusions

In this study, Ni-doped ZnO nanofibers were successfully deposited onto bidirectional carbon fabrics through electrospinning followed by calcination. The modified carbon fabric is subsequently incorporated into epoxy matrix to form Ni-ZnO nanofiber modified CFRP composite laminates. Structural characterization confirmed the formation of hexagonal wurtzite ZnO with successful Ni incorporation, while SEM observations revealed a uniform nanofiber distribution with nanoscale diameters that facilitated interfacial bonding with the carbon substrates. FTIR spectra further indicated the presence of Ni–O vibrational modes, validating the hybrid dielectric–magnetic nanostructure. Electromagnetic shielding measurements in the X-band (8–12 GHz) revealed that neat carbon fabric/epoxy laminates exhibited limited shielding, primarily due to reflection. The incorporation of Ni-ZnO nanofibers significantly enhances shielding effectiveness by introducing synergistic dielectric loss (from ZnO) and magnetic loss (from Ni), along with interfacial polarization and multiple scattering within the stacked architecture. The three-layer Ni-ZnO nanofiber-coated laminate achieved a maximum shielding effectiveness of ~ 85 dB, corresponding to an attenuation of more than 99.99% of the incident EM radiation, with absorption playing the dominant role. These results establish that Ni-ZnO nanofiber-modified carbon fabrics offer an effective strategy to overcome the limitations of conventional CFRPs in EMI shielding.

## Data Availability

All the data used in the article have been made available in the present article. The data that support the findings of this study are available from the corresponding author, Shivamurthy B upon reasonable request.
